# Inter-jurisdictional cooperation on pharmaceutical product listing agreements: views from Canadian provinces

**DOI:** 10.1186/1472-6963-13-34

**Published:** 2013-01-31

**Authors:** Steven G Morgan, Paige A Thomson, Jamie R Daw, Melissa K Friesen

**Affiliations:** 1 Centre for Health Services and Policy Research, School of Population and Public Health, Faculty of Medicine, University of British Columbia, 201-2206 East Mall, Vancouver V6T 1Z3, Canada

**Keywords:** Prescription drugs, Reimbursement mechanisms, Risk sharing, International cooperation, Canada

## Abstract

**Background:**

Confidential product listing agreements (PLAs) negotiated between pharmaceutical manufacturers and individual health care payers may contribute to unwanted price disparities, high administrative costs, and unequal bargaining power within and across jurisdictions. In the context of Canada’s decentralized health system, we aimed to document provincial policy makers’ perceptions about collaborative PLA negotiations.

**Methods:**

We conducted semi-structured telephone interviews with a senior policy maker from nine of the ten Canadian provinces. We conducted a thematic analysis of interview transcripts to identify benefits, drawbacks, and barriers to routine collaboration on PLA negotiations.

**Results:**

Canadian policy makers expressed support for joint negotiations of PLAs in principle, citing benefits of increased bargaining power and reduced inter-jurisdictional inequities in drug prices and formulary listings. However, established policy institutions and the politics of individual jurisdictional authority are formidable barriers to routine PLA collaboration. Achieving commitment to a joint process may be difficult to sustain among heterogeneous and autonomous partners.

**Conclusions:**

Though collaboration on PLA negotiation is an extension of collaboration on health technology assessment, it is a very significant next step that requires harmonization of the outcomes of decision-making processes. Views of policy makers in Canada suggest that sustaining routine collaborations on PLA negotiations may be difficult unless participating jurisdictions have similar policy institutions, capacities to implement coverage decisions, and local political priorities.

## Background

To avoid the negative effects of widely-used external reference pricing policies, manufacturers are placing tighter restrictions on list prices for prescription drugs [1-3]. Final drug prices are therefore increasingly determined by confidential contracts negotiated between drug plans and manufacturers [4,5]. The secrecy of negotiated price rebates effectively segments the market, allowing firms to price discriminate across payers. This gives payers an opportunity for savings but also creates new challenges related to negotiation and enforcement.

While there are several examples of inter-jurisdictional cooperation on health technology assessment (HTA) for drug coverage decision-making [6-8], there have been few instances of inter-jurisdictional cooperation on price negotiations. However, to increase purchasing power, reduce administrative costs, and prevent unwanted price disparities across payers, cooperation on price negotiation might be justified in some cases. Canada offers an illustrative example.

Canada differs from some other federal states, such as Italy or Australia, where pharmaceutical pricing policies are centralized with a national authority. Responsibility for health care in Canada is devolved to its ten provinces, which vary considerably in population size and income – see Table 1. Prescription drug coverage policy is highly decentralized as there is no federal funding incentives to promote consistency of public drug coverage across provinces [9]. As a result, each province independently operates limited public drug benefit programs. These programs vary widely in structure and account for 26 to 45% of prescription drug expenditures in their provinces, leaving a significant role for private financing through insurance and out-of-pocket payments. All provinces except Quebec cooperate on health technology assessment for public drug coverage decision-making [7]; however, they remain responsible for their own coverage decisions and price negotiations.

**Table 1:** **Statistics on Canada**’**s provinces**

	**Population(2011)**	**Gross domestic product per capita(CAD$,2010)**	**Total prescription drug expenditure per capita(CAD$,2011)**	**Share of total prescription drug expenditure financed by provincial government(2011)**
Canada (total)	34,482,779	$47,000	$788	38%
Ontario	13,372,996	$46,000	$785	43%
Quebec	7,979,663	$40,000	$912	33%
British Columbia	4,573,321	$44,000	$575	36%
Alberta	3,779,353	$70,000	$725	45%
Manitoba	1,250,574	$43,000	$710	34%
Saskatchewan	1,057,884	$60,000	$799	38%
Nova Scotia	945,437	$38,000	$985	34%
New Brunswick	755,455	$39,000	$937	26%
Newfoundland and Labrador	510,578	$55,000	$920	32%
Prince Edward Island	145,855	$34,000	$791	31%

Sources: Authors’ analysis of data from Canadian Institute for Health Information and Statistics Canada.

Price contracts for pharmaceuticals are known as product listing agreements (PLAs) in Canada. Prior to approximately 2006, they were seldom ever used by any province. Today, use of PLAs varies widely across provinces: they are virtually never used in Quebec and Newfoundland and Labrador; they are used for virtually all drugs covered by the public drug plans in Ontario and Manitoba; and they are used to varying degrees in other provinces.

In 2010, provincial premiers announced that a Pan-Canadian Purchasing Alliance would facilitate joint price negotiations among interested provinces [10]. Joint negotiations related to three drugs had taken place by mid-2012 [11]. Such collaboration indicates potential for joint PLA negotiations; however, it is not yet clear whether PLA collaboration can be sustained and applied universally to new medicines as is collaboration on HTA. We therefore sought to explore provincial policy makers’ views of the benefits and drawbacks of joint negotiations and the potential barriers to making such collaborations routine.

## Methods

After ethics review and approval, we conducted telephone interviews with purposefully selected provincial drug plan executives. In January 2012, we sent study invitations to each of the ten provincial drug plans. Invitations were sent to the most senior executive within each plan or, where identifiable, the executive responsible for contract negotiations. Invitees were asked to participate in a telephone interview or to identify an appropriate individual to speak on behalf of their jurisdiction. Nine provinces agreed to participate; only one province, Newfoundland and Labrador, declined.

Seven of the nine policy makers interviewed had worked with their respective drug plans for at least five years. The other two policy makers were hired more recently to manage new PLA negotiation processes in their jurisdictions. All policy makers from provinces that had used PLAs had direct experience with PLA negotiations and related inter-provincial collaboration efforts.

The confidential nature of PLAs meant that policy makers could not be asked to comment on particular negotiations or signed PLAs. Participants were therefore asked to describe the general benefits and drawbacks of collaboration on PLA negotiation and to provide their opinion about the barriers and facilitators to collaborating on a routine basis. Additional file 1 contains the interview guide.

Interviews lasted an average of 40 minutes (range 28 to 54 minutes), and were recorded and transcribed for thematic analysis [12]. All four authors read all transcripts and independently identified themes that emerged from the text. Authors met and jointly developed a coding scheme that then was used by two authors to independently code each transcript. Coding discrepancies were resolved through consensus or by consulting the lead author.

## Results

Table 2 lists the benefits, drawbacks and barriers to joint PLA negotiation most commonly mentioned by participating policy makers. Key themes are described here.

**Table 2:** **Most frequently mentioned themes concerning the benefits**, **drawbacks**, **and obstacles to collaboration on product listing agreements**

	**Number of policy makers that mentioned theme(n = 9)**
**Benefits**	
Increased bargaining power	7
Provincial equity in price	5
Provincial equity in access	5
Administrative efficiency	2
Increased access, can fund more drugs	1
**Drawbacks**	
Delay/length of process	5
Reduction in provincial autonomy	4
Savings may be lost for large provinces	2
Resource-intensive	2
Decreased access in some jurisdictions	1
**Barriers**	
Differences in policy institutions	6
Provincial and/or federal will	6
Differences structure of drug benefit programs	5
Technical and administrative resources	5
Political acceptability of the decision	4

### Benefits of collaboration

Policy makers from all participating provinces expressed general support for collaborative PLA negotiations. Seven of the nine policy makers participating in our study noted that the increased bargaining power achieved through collective negotiations would likely lead to lower prices and cost savings for participating jurisdictions (see Table 2). Several policy makers noted that this benefit would not be shared equally as price savings are likely to be greatest for the smaller provinces, which in isolation have lower bargaining power due to population size.

Five policy makers also explicitly identified interprovincial price consistency as a benefit of a joint negotiation process. Under the current system of independent negotiations, policy makers in several provinces expressed concerns that manufacturers may provide the first and most generous price concessions to one or two large provinces, increasing political pressure on other provinces to provide coverage. This strategy, known as whipsawing, could force small provinces – with limited negotiating capacity and purchasing power – to pay the highest prices. Policy makers from five provinces – large and small – stated PLA collaboration may be one way of addressing this issue and improving interprovincial equity in medicine prices and access. As a policy maker from one of the larger provinces stated, by working together, provinces could “*…break down barriers [to access] wherein depending on where you live, you may have different access to medications*” (Interviewee #6).

### Drawbacks from collaboration

Policy makers from five of the participating provinces expressed concerns that joint negotiations might increase the time it takes to list a drug on provincial formularies. They argued that multilateral negotiations, by nature of the number of stakeholders at the table, would require more time than bilateral negotiations. Two policy makers also noted that joint negotiations may require more resources overall because each jurisdiction would still need to bear the cost of evaluating and adapting PLAs for the local health system and policy framework.

Perceived loss of provincial autonomy was one the most cited drawbacks of joint negotiations. This included concerns about lines of accountability for PLA decisions that often have significant budget impacts. It also included considerations related to the ability of individual jurisdictions to respond to the varying politics of their jurisdiction, each with different political parties, priorities, and dominant interest groups. One policy maker noted that “*politicians have different pressures at different times*” (Interviewee #3) making it difficult to predict whether a jointly negotiated PLA will have local support.

### Barriers to expanded collaboration

#### Institutional barriers

Policy makers from six provinces mentioned institutional barriers to routine collaboration on PLA negotiations, ranging from constitutional authority over health care to local policy processes. Related to the former, several policy makers noted that provinces have independent authority for health care policy and related decision-making. It was argued that co-operation among the autonomous provinces is difficult without financial incentives from the federal government. Reflecting more local considerations, policy makers also noted that drug coverage decision-making processes vary considerably across provinces. While some provinces cede coverage decisions to executives responsible for public drug programs, others require ministerial or even cabinet approval. These differences affect decision-making timelines and consultation requirements in ways that might impede routine collaboration.

Policy makers from five provinces also cited institutional variations in provincial drug benefit structures as a barrier to routine collaboration on PLA negotiation. Given that some provinces provide comprehensive coverage for the elderly and the poor and others only cover residents against “catastrophic” drug costs, the coverage priorities, decision-making frameworks, and even bargaining positions will differ significantly from province to province. One policy maker went so far as to conclude that joint negotiations are unlikely to become commonplace unless all participating provinces offered comparable drug benefit plans: *“Fundamentally, you’ve got to have the same drug benefit plan before you actually negotiate together*” (Interviewee #5).

#### Political barriers

Political will was one of the most frequently mentioned obstacles to routine collaboration on PLA negotiation. Policy makers from several provinces felt that joint negotiations are likely to be explicitly or implicitly driven by larger provinces – because they have the greatest purchasing power and political influence – and that this would not sit well with some governments. Reflecting the constitutional autonomy mentioned above, some policy makers said that provinces would not voluntarily cede the decision-making authority required to sustain joint PLA negotiations. As one policy maker stated, “[y*ou have to] give one group authority to make decisions that would be binding on all provinces, which isn’t going to happen*” (Interviewee #7).

Related to the broader concerns about political will, policy makers from four provinces cited concerns about the local political acceptability of final listing decisions that would result from a joint negotiation. Two of these policy makers specifically noted that there is a risk of provinces circumventing the collective process on a drug-by-drug basis, either by pursuing individual negotiations or by “*cherry-picking*” only those jointly-negotiated PLAs that suit their interest. Though acting in the interest of one’s own jurisdiction is the primary responsibility of provincial policy makers, such outcomes were viewed as significant threats to the effectiveness and sustainability of routine joint negotiations.

#### Resource barriers

Policy makers from five of the participating provinces cited scarcity of required resources as a barrier to expanding joint PLA negotiations. As stated above, some policy makers felt that joint processes, at least currently, represent an increase in negotiation costs. Some provincial policy makers argued that the federal government should support the infrastructure required for collaboration among the provinces. They also noted that this was unlikely given the federal government’s current spending priorities and fiscal constraints.

## Discussion

Policy makers from Canadian provinces express general support for joint PLA negotiations. Despite support in principle, none suggested that routine collaborations would be sustainable at present because of variations in existing policy institutions and politics. This view might be surprising given the longstanding collaborations among all but one province on health technology assessment related to drug coverage decision-making [7]. However, a critical difference in these processes is that collaboration on health technology assessment harmonizes the *evidence* underpinning coverage decisions while collaboration on PLA negotiations effectively harmonizes the coverage *decisions*. To provide firms with maximum incentive to negotiate better deals than would be the case in independent negotiations, a PLA collaboration has to result in consistent funding decisions: failure to reach agreeable terms through joint negotiations must result in a “no” listing decision by all participating drug plans, and a successful joint negotiation must result in a “yes” decision. Achieving and sustaining such commitment to joint decision-making is a formidable challenge.

The insights provided to us by policy makers from Canadian provinces illustrate that joint negotiations may break down for a number of reasons. Acting in self-interest, payers may perceive that a better (or faster) deal can be achieved by negotiating independently or may resort to “cherry picking” their commitment to joint negotiations. Manufacturers may even have incentive to try to break a negotiation alliance by offering side deals in the hope that individual drug plans could be played against each other.

Beyond these self-interested incentives to defect, policymakers expressed greater concern that solidarity in negotiations would break down as a result of institutional and political barriers. Willingness to implement consistent funding decisions through joint negotiations requires, at least, that participating drug plans have comparable decision-making frameworks and negotiating leverage. Differences in policies as simple as decision-making protocols can limit the potential for routine collaboration. Variations in the benefit structure of drug programs – that is, who is covered and with what types of user charges-are even more important because they may be more costly to harmonize and they have a more profound effect on decision-making frameworks. This raises the broader issue of the politics of decision making autonomy and accountability.

Being committed to the decision-making outcomes associated with joint PLA negotiations requires that decision-making autonomy be ceded to a collective process. This requires a great deal of political will because different governments have different health care priorities and competing interests, such as attracting or retaining pharmaceutical industry investment. It was not stated explicitly by any policy makers in our study, but variations in provincial incomes and, therefore, governments’ revenue generating powers will also affect the acceptability of centralized decisions. Given the significant economic disparity among negotiation partners, some participating jurisdictions may simply not be able to “afford” to commit to collective decisions.

### Limitations

Our study is not without limitations. First, we only interviewed one policy maker in each province. These policy makers were senior executives and managers with direct experience related to PLA negotiations and related collaborative efforts, or direct experience with drug coverage decision-making if PLAs were not used in their province. Their views are those of people closely engaged in the file but will not necessarily reflect views of other officials, such as ministers or deputy ministers of health. As with any study of this kind, the views expressed by respondents represent a snapshot of perceptions in a complex policy arena. Policy makers’ perceptions will evolve as provinces’ gain more experience with PLAs and related cooperative efforts. Finally, we are aware that interview responses may have been influenced by social desirability bias. In an effort to mitigate that bias, participants were informed that our study would not attribute themes or quotes to specific provinces or speakers unless absolutely necessary and only after consent was provided.

## Conclusions

Joint negotiation of PLAs may be seen as a logical extension of inter-jurisdictional cooperation on health technology assessment; however, the extension is arguably a significant leap, one requiring a move from collaboration simply on decision-making processes to collaboration on decision-making outcomes. Canada’s experience suggests that building and sustaining a PLA negotiation alliance will likely require more than simple commitment to avoid defection due to self-interest. Differences in policy institutions, political pressures, and economic opportunities of participating jurisdictions can place considerable strain on a joint negotiation process.

Choice of “negotiating partner” is a key determinant of effective, routine collaboration on PLA negotiations. Sustaining routine collaborations on a voluntary basis will require partners with similar drug plans, compatible priorities for health care and industrial development, and comparable income levels. Whether across provinces in Canada or across countries internationally, such collaborations would address some of the shortcomings of by increasing purchasing power and administrative efficiencies among negotiation partners. They would not, however, address concerns about global disparities in drug prices that might arise in the new paradigm of confidential drug pricing.

To promote equity in negotiation processes and outcomes in a federation such as Canada, it would be possible for the federal government to either centralize this policy file as is done in countries like Italy and Australia, or to actively encourage collaborations among disparate states/provinces. Given the politics of jurisdictional responsibility in Canada, centralization is unlikely. The Canadian government could, however, exercise its spending power to encourage collaboration. By sharing part of the cost of the negotiation process and related funding decisions, conditional on provinces harmonizing the structures of drug benefit programs, it could incentivize participation while equalizing decision-making frameworks and addressing income disparities that limit voluntary efforts by provinces.

While centralization by way of vertical policy integration is possible in the context of a federation, it is not possible across countries. It will therefore be more difficult to improve equity in drug pricing across nations with disparate income levels. If the new global pricing paradigm is resulting in increasingly inflated list prices for medicines, mechanisms will be needed to assist less wealthy countries in negotiations, co-ordinated through agencies such as the World Health Organization.

## Abbreviation

PLA: Product listing agreement.

## Competing interests

Steven Morgan has been retained as a consultant on matters related to pharmaceutical policy by Health Canada, the Department of Justice Canada, and the British Columbia Ministry of Health Services. The other authors have no conflicts of interest to declare.

## Authors’ contributions

SM is responsible for project conception and acquisition of funding. All authors contributed to the study design. SM and PT conducted the telephone interviews. All authors contributed to the thematic coding of interview transcripts. SM wrote the first draft of the paper. All authors revised and provided comments on paper drafts and have approved the final version of this paper.

## Pre-publication history

The pre-publication history for this paper can be accessed here:

http://www.biomedcentral.com/1472-6963/13/34/prepub

## Supplementary Material

Additional file 1 **Appendix 1.** Interview Guide. Click here for file
