# Supplementary material for: Inter-jurisdictional cooperation on pharmaceutical product listing agreements: views from Canadian provinces
Source: BMC Health Serv Res. 2013 Jan 31;13:34. doi: 10.1186/1472-6963-13-34 (PMC3563436; doi:10.1186/1472-6963-13-34)
Supplement: Additional file 1 — Appendix 1. Interview Guide. [file 1472-6963-13-34-S1.docx]

# Appendix 1: Interview Guide

*Our research project is focused on the extent of and issues related to the use of supply contracts (also called product listing agreements or reimbursement schemes) between drug manufacturers and organizations that fund prescription drug purchases in several countries around the world. Contracts we are interested in include any negotiated agreement that affects the net price paid for a medicine, including agreements regarding how prices will be affected by utilization levels, utilization patterns, or health outcomes.*

*In recent years, it has been proposed that Canadian provinces co-operate (either independently or with the federal government) to establish a joint purchasing mechanism for prescription drugs.*

1. What would a joint purchasing mechanism look like in your view?
2. What do you see as the potential benefits of a joint purchasing mechanism?
3. What do you see as the potential downsides of a joint purchasing mechanism?
4. *To date, there has been limited action towards establishing a joint purchasing mechanism for prescription drugs.* What are the key obstacles to establishing such a mechanism?
5. What do you think it would take for such a joint purchasing mechanism to be implemented and applied broadly (e.g., for purchases of many if not all medicines)?
